# Comparison of breath-guards and face-masks on droplet spread in eye clinics

**DOI:** 10.1038/s41433-022-02308-8

**Published:** 2022-12-03

**Authors:** Richard Newsom, Chris Pattison, Andrew Lundgren, Pauline Robison, Matthew Quint, Adam Amara

**Affiliations:** 1 School of Health and Care Professions, University of Portsmouth, Portsmouth, UK; 2 Institute of Cosmology and Gravitation, University of Portsmouth, Portsmouth, UK; 3 Department of Optometry, University of Portsmouth, Vision Science, Portsmouth, UK; 4 Gravitational Wave Physics, Institute of Cosmology and Gravitation, University of Portsmouth, Portsmouth, UK; 5 Portsmouth Hospitals University Trust, Portsmouth, UK

**Keywords:** Risk factors, Microbiology

## Abstract

**Introduction:**

COVID-19 has impacted ophthalmic care delivery, with many units closed and several ophthalmologists catching COVID-19. Understanding droplet spread in clinical and training settings is paramount in maintaining productivity, while keeping patients and practitioners safe.

**Objectives:**

We aimed to assess the effectiveness of a breath-guard and a face mask in reducing droplet spread within an eye clinic.

**Methods:**

We performed a randomised trial of droplet spread using a fluorescein-based cough model to assess the efficacy of a ‘breath-guard’ and ‘face-mask’ to prevent the spread of droplets. The ‘cough’ spray was collected on calibrated paper targets. The sheets were photographed under blue light, with an orange filter on the camera; the position and size of the spots was measured with software originally developed for astronomy.

We performed 44 randomised coughs; 22 controls with no breath-guard or face-mask, 11 using breath-guard only and 11 with combined breath-guard and face-mask. We compared both the number of droplets detected and the area of drops on paper targets.

**Results:**

The average number of droplets in the controls was 19,430 (SE 2691), the breath-guard group 80 (SE 19) droplets (*P* < 0.001); in the combined In the group the count was 5 (SE 2), a significant drop from shield only (*P* = 0.008). The mean areas of each target covered by spots for each group were 5.7 ± 0.857% (95% CI), 0.004 ± 0.000104% (95% CI) and 0.001 ± 0.0000627% (95% CI) respectively.

**Conclusion:**

These results show that the breath-guard alone reduced the droplet count by 99.93%. Combining the breath-guard with a face-mask reduced the droplet count by over 99.98%. Breath-guards are widely used in clinics and this trial demonstrates that breath-guards with face-masks effectively block droplet spray.

## Introduction

Transmission of COVID-19 within ophthalmology clinics is recognised as a significant danger to both patients and staff members. The sustained close proximity of patients to healthcare workers in enclosed (and sometimes poorly ventilated) ophthalmology clinics leads to high levels of droplet and aerosol contamination [1]. This is reflected in a high rate of cross infection and the death of several ophthalmologists during the pandemic [2–4].

Evidence suggests that person-to-person transmission occurs through droplets, or aerosols, of infected saliva and respiratory secretions when a person coughs, sneezes or talks [5–7]. Jones et al. [8] had previously reported that larger respiratory droplets, such as those expelled for a cough, would fall quicker than smaller aerosolized particles from speech due to gravitational forces. However, some now consider droplets up to 100 μm as aerosols, and others have shown that a cough can propel droplets of these sizes for over 8 m [9, 10]. A recent trial by our group, using a cough model, has shown that droplets regularly travel further than 2 m even within a laminar flow operating theatre [11].

The use of ophthalmic equipment, such as the slit lamp biomicroscope or direct ophthalmoscope, require close working distances. Common transmission routes for COVID-19 are from droplets or aerosols, via the eye [12–15] or from fomite spread [16]. Ophthalmologists and other eye care health professionals, who undertake face-to-face patient clinical examinations, are therefore at a heightened risk of transmission of the COVID-19 virus [17].

Patient infection risk may also be great, and it should be recognised that 25–30% of all COVID-19 deaths are thought to have originated from health care workers, many of whom are asymptomatic [18, 19]. This indicates the need for precautionary measures within ophthalmic departments, who treat mainly elderly patients, and other eye care practices to minimise risk of transmission whilst undertaking ophthalmic investigations [20].

To mitigate these risks, patients have been encouraged to wear face-masks and health care workers have worn PPE. Other methods include increased ventilation and physical barriers or ‘breath-guards’ to prevent a direct spray between patient and staff [21].

The Portsmouth group has developed a number of methods for generating a cough model, and detecting the spray of droplets in different clinical situations. We stain droplets with fluorescein, and use forensic imaging techniques to photograph the droplets. Droplets were counted using image analysis techniques in the Institute of Cosmology and Gravitation.

We performed a randomised controlled trial of a control group with no shield vs ‘breath-guard’ vs ‘breath-guard and face-mask’ to measure the effectiveness of the breath-guard on droplet contact with the clinician. In the remainder of this paper, we refer to the ‘breath-guard and face-mask group’ as simply the ‘breath-guard group’.

## Methods

**Fig. 1:**
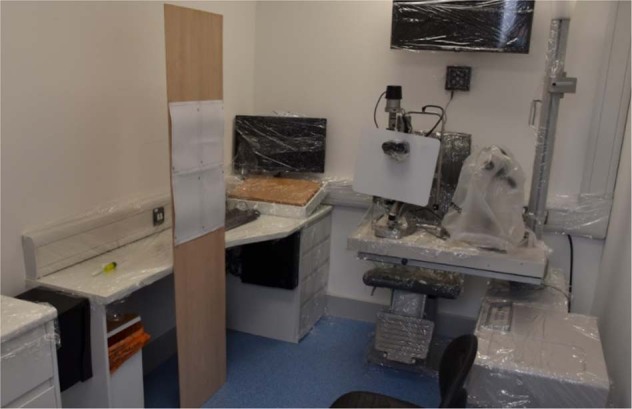
This is the  experimental setup, the paper targets are pinned to a board and the breath-guard is in place.

We used a cough model, taped to a slit lamp in the University of Portsmouth Eye Clinic. The cough model uses an Ambu bag connected to 40 cm of 30 mm endotracheal tubing. We calibrated this to give a FEV1 between 290 and 370 l/mm, similar to a human cough. To produce a ‘dry’ cough we used 0.5 ml of normal saline, stained this with 1 mg/100 ml fluorescein (Fig. 1).

**Fig. 2:**
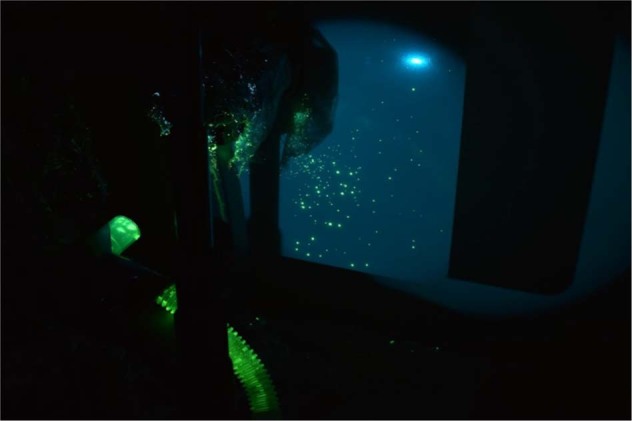
An example of a splatter pattern and no breath-guard.

We used a randomised controlled trial method, random numbers were used to determine whether the breath-guard, breath-guard with a face-mask or control. The breath-guard we used was 300 mm high and 350 mm wide and was attached to the eye pieces of the slit lamp (Fig. 2).

We placed a target of four A4 printed sheets on a board at 40 cm (the standard position of the observer eyepieces) from the cough model, behind the slit lamp and triggered the ‘cough’. The targets were imaged under UV light in a dark room, using a blue blocking filter (Tiffen, New York, Orange 16). Sets of images were taken with the control (22), the breath-guard (11) with the breath-guard and face-mask (11).

**Fig. 3:**
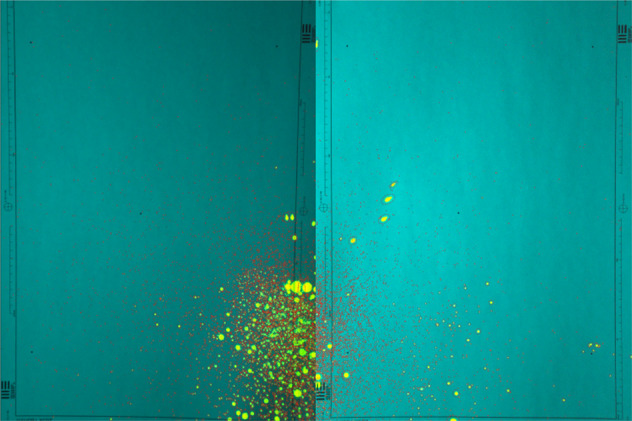
This figure shows the software detection of droplets on the target paper sheets, each droplet is circled in red, before the size, brightness is measured.

The images were first de-warped to correct the camera angle, and a source detection algorithm, Source Extractor [22], was then used to detect droplet ‘spots’ on the targets. The algorithm identified spots that were an area of 5 pixels or larger, which corresponds to a physical area of approximately 40 μm^2^ (Fig. 3). We compared the total numbers of spots for each group, as well as the total area of splatter on each target.

This research was submitted to the University of Portsmouth Ethics screening tool ref: 10247.

## Results

**Fig. 4:**
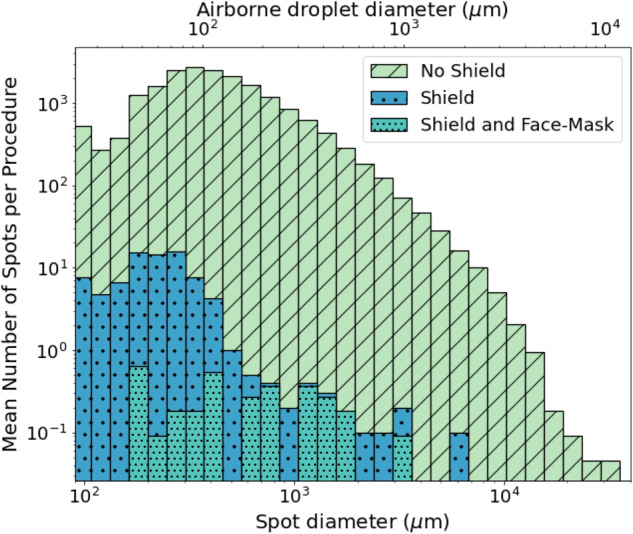
Histogram of average number of droplets vs size for control, breath-guard, and face-mask with a breath-guard.

We detected 19,430 (SE 2,691) droplets in the control group; 80 (SE 19) in the breath-guard (*p* < 0.001), and 5 (SE 2) in the face-mask group (*P* = 0.008). (Fig. 4). The average area of each target covered by the droplets in the control group was 5.7 ± 0.857% (95% CI), 0.004 ± 0.000104% (95% CI) in the breath-guard group (95% CI 0.02, 0.001), and 0.001 ± 0.0000627% (95% CI).

Using the mean area of the targets covered in spots as a measure, there was a 99.93% reduction in droplet spread with breath-guard and a 99.98% with the face-mask and breath-guard group. We also noted fluorescein splattered on the floor and on the researchers, this was invisible to the human eye but illumination under UV light revealed the spots within the environment.

## Discussion

The results show the sensitivity of this technique in detecting droplets, and the number of droplets that a single cough can generate, with the potential of COVID-19 infection within an eye clinic. This has been suggested in other clinical environments [9], however this is the first time it has been demonstrated within an eye clinic.

The breath-guard effectively reduced the spread of droplets from the cough model, this effect was enhanced using a face-mask. We found a 99.93% fall in the area of spots detected with a breath-guard and 99.98% reduction with the face-mask as well as the breath-guard. However, some drops still escaped and as only one droplet may be sufficient to transmit COVID-19 other measures to prevent droplet transmission are clearly still vital. These results are consistent with the droplet spread found in other healthcare environments. A physical barrier may be key to prevent droplet spread. This is clearly an important feature of safety within the ophthalmology clinic for slit lamp users but for other equipment as well (such as autorefractors and biometry).

However, the breath-guards are not protective against the smallest droplets such as aerosols [23], these will remain airborne for many minutes, and may affect the physician as well as the patient and subsequent patients. Good ventilation is important as well and the air turnover is recommended at 10 cycles per minute [24]. This is a great change for most eye clinics, many of which are situated in unventilated areas of the hospital.

One last consideration was that the fluorescein actively stained fluid that was detected on the floor, chair, swabs and on the instruments. This was of considerable surprise to the ophthalmologist. Use of fluorescein in this way may be a useful clinical test for fomite transfer [25] within an eye clinic and to monitor cleaning [26].

The limitations of this trial were that it was not possible to accurately measure the aerosol release and as these play a vital role in the propagation of disease [27] it was not possible to fully understand the effectiveness of the breath-guard. It may also be useful to assess other sizes of breath-guard and types of face-masks [28, 29]. Other areas of interest would be the effect of smaller cough volumes, the effect of face-masks [30] and the effect of ventilation / air filtration equipment.

Given the relative size of the droplets the volume of virus carried in these is large and protection against droplets plays an important role within the eye clinic.

## Conclusion

Larger breath-guards significantly reduced the droplets spread, with the addition of face-masks very few droplets spread from the patient. Use of face-masks and breath-guards play an important role in reducing the viral load spread between the clinician and the patient. Smaller guards may not be as effective. It would be useful to test the model on patient volunteers and to assess the effect of aerosols and ventilation on respiratory virus transmission, within eye clinics.

## Summary

### What was known before

- Ophthalmologist and optometrist were at risk of catching COVID-19.

### What this study adds

- A breath-guard reduces the spread of droplets by 99.8% within eye clinics. The fluorescein and Astrophysics software to analyse droplet spread in a clinical environment was described.
